# A two-year longitudinal study of retinal vascular impairment in patients with amnestic mild cognitive impairment

**DOI:** 10.3389/fnagi.2022.993621

**Published:** 2022-11-07

**Authors:** Chiara Criscuolo, Gilda Cennamo, Daniela Montorio, Antonio Carotenuto, Miriana Migliaccio, Marcello Moccia, Elena Salvatore, Roberta Lanzillo, Ciro Costagliola, Vincenzo Brescia Morra

**Affiliations:** ^1^Reproductive and Odontostomatological Sciences, Department of Neurosciences, University of Naples “Federico II”, Naples, Italy; ^2^Public Health Department, Eye Clinic, University of Naples “Federico II”, Naples, Italy

**Keywords:** OCTA, aMCI, Alzheimer’s disease, longitudinal study, mini mental state examination

## Abstract

**Objective:**

To evaluate the relation between retinal vascular impairment and cognitive decline in patients with amnestic mild cognitive impairment (aMCI) over time.

**Methods:**

Spectral domain-optical coherence tomography (SD-OCT) and OCT angiography study was performed in aMCI patients over 2 years follow-up and compared to baseline.

**Results:**

Thirty-eight eyes from 19 aMCI patients were evaluated. Structural and vascular OCT measures were reduced at follow-up except for vessel density (VD) of the choriocapillaris, unchanged, and foveal avascular zone, which was increased; no changes in any parameter were found in 18 age-matched healthy controls. Overall, these findings were confirmed when patients were evaluated separately according to progression to dementia. Only non-converters to dementia showed significant VD reduction in the deep capillary plexuses (coeff. β = −4.20; *p* < 0.001), may be for an initial massive VD depletion becoming less evident with progression of the disease. MMSE reduction was associated with a higher ganglion cell complex reduction (coeff. β = 0.10; *p* = 0.04) and a higher VD reduction in the radial peripapillary capillary (RPC) plexus (coeff. β = 0.14; *p* = 0.02) in the whole patient group, while it was associated with a higher VD reduction only in RPC plexus in converters (coeff. β = 0.21; *p* < 0.001).

**Conclusion:**

Our data shows vascular impairment progression in the inner retina of aMCI patients and support the hypothesis that vascular changes may contribute to the onset and progression of Alzheimer’s disease. Other follow-up studies, with a larger number of patients, are needed to better define VD as a potential biomarker.

## Introduction

Mild cognitive impairment (MCI) is considered the transitional stage between physiological age-related memory decline and dementia.

MCI, especially amnestic MCI (aMCI), is a risk factor or a prodromal stage of Alzheimer’s disease (AD) (Petersen, 2011; Boeve, 2012). Early intervention, for example in the MCI stage, is then crucial for the future control of this neurodegenerative disease, because brain pathology changes are found years or even decades before the cognitive and functional decline in AD patients (Petersen, 2011; Lin et al., 2014; Chiang et al., 2021; Guzman-Martinez et al., 2021). Therefore, it is imperative to find clinically feasible biomarkers that can predict or monitor the course of MCI (Chiang et al., 2021; Liss et al., 2021).

The eye, allowing for non-invasive imaging of neural tissue as well as of its microcirculation, represents an interesting field of biomarkers research (Frost et al., 2010).

Optical coherence tomography angiography (OCTA) is a non-invasive, rapid and efficient imaging tool able to detect vascular network changes in aging population, in MCI and AD patients (Ascaso et al., 2014; Chua et al., 2020; Sardone et al., 2021; Giuliani et al., 2022; Niro et al., 2022).

Morphological and functional alterations of cerebral blood vessels have been reported in AD (Ries et al., 1993; Schuff et al., 2009; Snyder et al., 2014) and these vascular changes are reflected in the retina due to the homology between retinal and cerebral vasculature.

Whether these structural and vascular changes in the eye may be suitable as a biomarker for detection and follow-up of MCI or AD is still a matter of controversy.

Therefore, to explore retinal vessel pattern in cognitive decline in 2020 we performed a cross-sectional baseline study in order to evaluate OCT parameters in aMCI patients vs. healthy subjects (Criscuolo et al., 2020). Here, to explore if retinal structural and vessel features can be useful as indicators of disease progression, we conducted a two phase prospective study in the same aMCI patients 2 years later. We evaluated how OCT parameters varied over time and correlated OCT parameters with cognitive impairment progression.

## Materials and methods

In this prospective study, we performed a complete neurological and ophthalmological examination after 2 years of follow up in our previously reported cohort of aMCI patients (Criscuolo et al., 2020).

MCI participants initially were revaluated and diagnosed clinically by experienced neurologists with a specialization in memory disorders (CC and ES) based on the clinical guidelines and recommendations of the National Institute on Aging-Alzheimer’s Association (Albert et al., 2011). Clinical history, cognitive testing and neuroimaging were reviewed for diagnostic accuracy by a multidisciplinary meeting that included neurologists, psychiatrists, and neuropsychologists to discussed and confirm the diagnosis of MCI or the progression to dementia according to preservation or not of activities of daily living.

At baseline, Β-Amyloid targeted positron emission tomography (PET) imaging was performed in a sub group of six patients. Due to pandemic only one more patient underwent Β-Amyloid PET during the follow up period.

Cognitively normal controls were enrolled and cognitively revaluated during the same study period from the control cohort already reported (Criscuolo et al., 2020).

A total of 10 patients and 11 healthy subjects had controlled hypertension.

During enrollment, all, patients and controls, underwent systemic blood pressure measurements that turned to be within the normal range.

A complete ophthalmological examination was performed in each subject including evaluation of best-corrected visual acuity according to Early Treatment of Diabetic Retinopathy Study, slit-lamp biomicroscopy, fundus examination with a + 90 D lens, spectral domain (SD)-OCT and OCTA.

Inclusion criteria included normal ophthalmic examination and normal intraocular pressure.

Exclusion criteria were: the presence of congenital eye disorders, myopia greater than six diopters, history of intraocular surgery, presence of significant lens opacities or any macular disease, previous diagnosis of glaucoma, evidence of vitreoretinal disease, uveitis and diabetic retinopathy.

Patients with a history of other neurological or psychiatric disorders, history of stroke, coagulopathy, diabetes, uncontrolled hypertension, head trauma, alcohol or drug addiction, or depression were also excluded. The study was approved by the Institutional Review Board of the University of Naples “Federico II” (protocol number: 142/19) and all investigations adhered to the tenets of the Declaration of Helsinki. Signed informed consents were obtained from each subject.

### Optical coherence tomography

Retinal nerve fiber layer (RNFL) and ganglion cell complex (GCC) thickness were analyzed by SD-OCT (software RTVue XR version 2018.1.1.60, Optovue Inc., Fremont, CA, United States). The circumpapillary RNFL was measured using a 3.45 mm radius ring centered on the optic disc. The GCC thickness was measured from the internal limiting membrane to the outer boundary of the inner plexiform layer. The scan was placed 1-mm temporal to the fovea over the macular region (7 × 7 mm^2^ area) (Hirashima et al., 2013).

### Optical coherence tomography angiography

OCTA images were obtained by the Optovue Angiovue System (software ReVue XR version 2018.1.1.60, Optovue Inc., Fremont, CA, United States). The software is based on a split-spectrum amplitude de-correlation algorithm which uses blood flow as intrinsic contrast (Jia et al., 2012).

The retinal vascular networks, superficial capillary plexuses (SCP), deep capillary plexuses (DCP), and choriocapillaris (CC) were evaluated in a 6 × 6 mm^2^ scan centered on the fovea.

The vessel density (VD) that corresponds to the percentage area occupied by the large vessels and microvasculature in the analyzed region, was automatically calculated by AngioAnalytics™ software (Huang et al., 2016).

Angiovue software automatically calculated the fovea avascular zone (FAZ) area over the 6 mm x 6 mm macular area in the full retinal plexus.

The VD of radial peripapillary capillary (RPC) plexus was analyzed with a scanning area of 4.5 × 4.5 mm^2^ area over the optic disc (whole image) from the inner layer membrane to the retinal nerve fiber layer posterior boundary (Rao et al., 2016).

The projection artefacts were removed by the 3D Projection Artefact Removal (PAR) algorithm in order to improve the depth resolution on OCTA signal and then distinguish vascular plexus-specific features. In particular, 3D PAR removed the projection of large vessels of the SCP on the DCP and CC (Zhang et al., 2016; Wang et al., 2017; Patel et al., 2018).

The analysis excluded OCTA images that presented a Signal Strength Index less than 80 and residual motion artefacts.

### Neuropsychological assessment

Cognitive functions were assessed in all subjects using an extensive neuropsychological battery, administered by two trained neuropsychologists. The battery included the Mini Mental State Evaluation (MMSE) (Folstein et al., 1975), for a general cognitive evaluation, and specific tests for several cognitive domains: (1) *Long*-*term memory*: Immediate and Delayed recall of a 15-Word List (I-Re and D-Re), Short Story Recall (SSRe), Delayed recall of Complex Rey’s Figure (Spinnler and Tognoni, 1987; Carlesimo et al., 1996, 2002); (2) *Short*-*term memory*: Digit span and Corsi Block Tapping task (Orsini et al., 1987); (3) *Language*: Token Test (De Renzi and Vignolo, 1962; De Bleser et al., 1986); (4) *Reasoning*: Raven’s Coloured Progressive Matrices (Carlesimo et al., 1996); (5) *Executive functions and attention:* Phonological Word Fluency (Carlesimo et al., 1996), Categorical Word Fluency, Attentional Matrices (Carlesimo et al., 1996); (6)*Praxis*: Copy of drawings (Spinnler and Tognoni, 1987; Carlesimo et al., 1996), Copy of Complex Rey’s Figure (Carlesimo et al., 2002). Adjustments for sex, age and education were applied according to Italian normative data.

### Statistical analysis

We described demographic, clinical, OCT and OCTA features using mean, standard deviation (SD), median and range. We tested normality for continuous variables through Shapiro–Wilk test and performed a between-group comparisons for demographic, clinical and structural MRI variables through t-Test, Mann–Whitney U or Chi-squared. Stata software (version 13; StataCorp LP, College Station, TX) was employed for statistical analyses.

We assessed SD-OCT and OCTA changes over the follow-up in patients and healthy groups through linear mixed models, including age, sex, disease duration at baseline as covariates and time points as factor of interest and either SD-OCT or OCTA measures as dependent variable. We also separately assessed SD-OCT and OCTA changes over the follow-up according to conversion to dementia using the same model. Finally, we assessed the association between MMSE changes (delta MMSE = MMSE at follow-up – MMSE at baseline) and both delta OCT and delta OCTA (follow-up – baseline) through a linear mixed models, including age and sex, as covariates, delta MMSE as factor of interest and either delta SD-OCT or delta OCTA measures as dependent variable. All statistical models here employed included subject as random factor, thus accounting for the within-subject inter-eye correlation. A *p* value <0.05 was considered statistically significant.

## Results

### Longitudinal clinical features and SD-OCT/OCTA findings in aMCI subjects

Nineteen patients (10 females, 9 males; mean age 75 ± 5.6 years) and 18 healthy controls (9 females, 9 males; mean age 75 ± 6.2 years) were enrolled. Of the 27 patients reported in the previous study two developed cataracts and one had COVID19, and therefore, we decided to exclude them. Five patients were lost at follow up, or declined to carry out a clinical evaluation at a hospital during the pandemic.

The two groups did not differ significantly in terms of age and gender. Demographic and clinical characteristics are summarized in Table 1.

**Table 1:** Demographic and clinical characteristics of cognitive healthy subjects, all patients, non-converters and converters to dementia after 2 years follow-up.

	HS	Patients	*p*-value	Non-converters	Converters	*p*-value
Subjects (n.)	18	19	–	12	7	–
Eyes (n.)	36	38	–	24	14	–
Age (years)	75 ± 6.2	75 ± 5.6	0.975	76.5 ± 5.4	76.3 ± 6.3	0.91
Sex (female/male)	9/9	10/9	0.872	7/5	3/4	0.36
MMSE score	–	23.1 ± 4.4	–	25.8 ± 2.2	18.8 ± 3.4	<0.001
Disease duration (months)	–	65 ± 24.8	–	61.2 ± 23.6	70.4 ± 27.2	0.18
Education	8.3 ± 2.5	9.3 ± 4.3	–	8.9 ± 4.6	10 ± 4	0.35

HS, healthy subjects; MMSE, mini mental state examination.

*p* value < 0.05.

Among the nineteen patients, seven (37%) patients converted to dementia. None reverted from aMCI to normal cognitive functions. Demographic, clinical characteristics according to conversion to dementia over the follow-up are summarized in Table 1. Patients converting to dementia at follow-up showed a lower MMSE at baseline compared to not converting patients (*p* < 0.001). Β-Amyloid targeted positron emission tomography (PET) imaging performed in seven patients was positive in five. These five amyloid-PET positive patients converted to dementia during follow-up.

**Figure 1:**
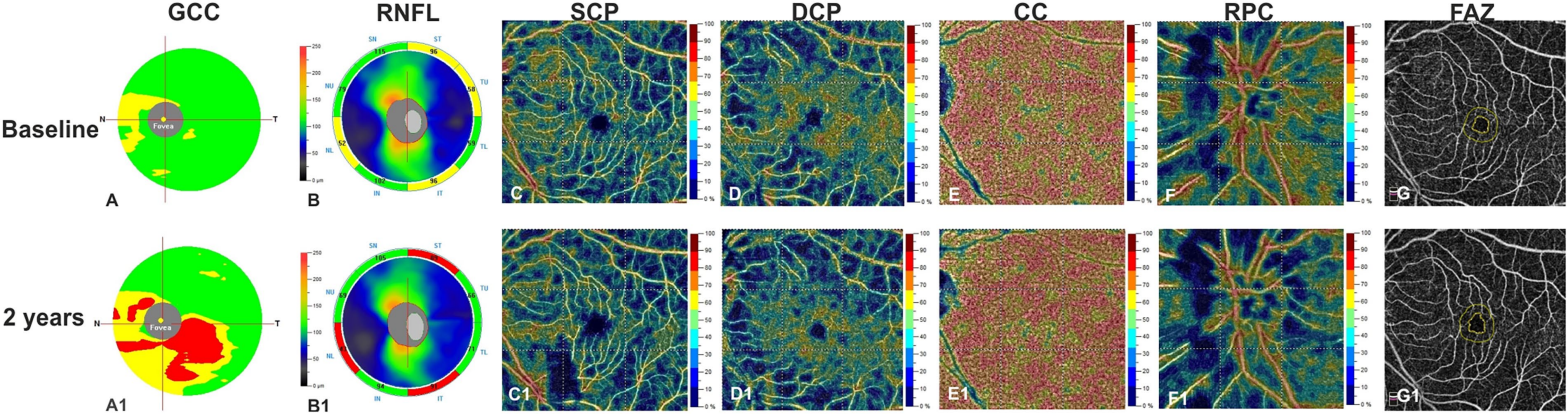
Left eye of a patient affected by MCI (72 years-old male) shows at structural spectral domain- optical coherence tomography (SD-OCT) an increased reduction in ganglion cell complex (GCC) **(A1)** and in retinal nerve fiber layer (RNFL) **(B1)** respect to baseline **(A,B)**. OCT Angiography images reveals a relevant vessel density reduction for superficial capillary plexus (SCP) **(C1)**, deep capillary plexus (DCP) **(D1)**, and radial peripapillary capillary plexus (RPC) **(F1)** at year 1 respect to baseline **(C,D,F)**. Vessel density in the choriocapillaris (CC) does not change over the follow-up (**E**,**E1**). The Foveal Avascular Zone (FAZ) area reveals an increased size at follow up **(G1)** respect to baseline **(G)**.

OCT and OCTA parameters revealed no significant changes after 2 years in healthy controls. OCT showed reduction of GCC and RNFL thicknesses over time (Estimated Mean Difference [EMD] = −4.21, 95% Confidence Interval [CI] = −6.55/−1.89, Cohen’s *d* = 0.44, *p* < 0.001; EMD = −4.47, 95%CI = −6.23/−2.71, Cohen’s *d* = 0.45, *p* < 0.001, respectively) in patients (Table 2). OCTA analysis showed VD reduction in SCP (EMD = −3.09, 95%CI = −4.46/−1.71, Cohen’s *d* = 0.65, *p* < 0.001), DCP (EMD = −3.37, 95%CI = −5.38/−1.36, Cohen’s *d* = 0.55, *p* = 0.001) and RPC plexus (EMD = −3.71, 95%CI = −4.43/−2.98, Cohen’s *d* = 0.69, *p* < 0.001), as well as a FAZ area increase (EMD = 0.11, 95%CI = 0.8/0.15, Cohen’s *d* = 0.99, *p* = <0.001) in patients, after2 years, compared to baseline. Conversely, VD of CC did not show significant differences between baseline and 2 years of follow up (Table 2; Figure 1).

**Table 2:** Comparison in SD-OCT and OCTA parameters among HS and all patients at baseline vs. follow-up.

	Baseline		Follow-up		HS follow-up vs.baseline		Patients follow-up vs. baseline	
	HS	Patients	HS	Patients	EMD	*P*-value	EMD	*P*-value
SD-OCT (μM)								
GCC average	98.53 ± 6.46	92.2 ± 9.5	97.38 ± 7.34	88.0 ± 9.5	−1.03	0.847	−4.21	<0.001
RNFL average	102.08 ± 7.94	95.9 ± 8.5	101.02 ± 8.93	92.1 ± 8.4	−1.11	0.764	−4.47	<0.001
OCTA (%)								
SCP whole	48.32 ± 4.28	45.3 ± 4.6	48.11 ± 3.25	42.3 ± 4.6	−0.12	0.831	−3.09	<0.001
DCP whole	50.25 ± 4.43	45.5 ± 6.2	50.01 ± 4.23	42.2 ± 5.7	−0.21	0.952	−3.37	0.001
CC whole	72.13 ± 4.19	70.1 ± 4.4	71.82 ± 4.61	69.8 ± 4.8	−1.14	0.852	−0.26	0.53
RPC whole	49.37 ± 5.88	46.5 ± 5.1	48.85 ± 5.27	42.9 ± 5.3	−1.38	0.744	−3.71	<0.001
FAZ area	0.208 ± 0.06	0.3 ± 0.1	0.213 ± 0.05	0.4 ± 0.1	0.12	0.861	0.11	<0.001

HS, healthy subjects; SD-OCT, spectral domain optical coherence tomography; EMD, estimated mean difference; GCC, ganglion cell complex; RNFL, retinal nerve fiber layer; OCTA, OCT angiography; SCP, superficial capillary plexus; DCP, deep capillary plexus; CC, choriocapillary plexus; RPC: radial peripapillary capillary; FAZ: foveal avascular zone.

Linear mixed models, *p* value < 0.05.

### Correlation between OCT parameters and cognitive decline

When patients were divided in two groups according to the occurrence of conversion to dementia over the follow-up, we showed that both groups presented with a statistically significant thicknesses reduction of GCC (converters: EMD = −4.79, 95%CI = −7.35/−2.23, Cohen’s *d* = 0.23, *p* < 0.001; non-converters: EMD = −3.80, 95%CI = −7.35/−0.25, Cohen’s *d* = 0.13, *p* = 0.04) and RNFL (converter: EMD = −4.71, 95%CI = −7.29/−2.14, Cohen’s *d* = 0.05, *p* < 0.001; non-converter: EMD = −4.30, 95%CI = −6.69/−1.91, Cohen’s *d* = 0.12, *p* < 0.001) over the follow-up, compared to baseline (Table 3). Similarly, both patients groups showed VD reduction in SCP (converter: EMD = −2.93, 95%CI = −4.99/−0.89, Cohen’s *d* = 0.20, *p* = 0.005; non-converter: EMD = −3.19, 95%CI = −5.04/−1.34, Cohen’s *d* = 0.24, *p* = 0.001), and RPC plexus (converter: EMD = −4.40, 95%CI = −5.23/−3.57, Cohen’s *d* = 0.16, *p* < 0.001; non-converter: EMD = −3.22, 95%CI = −4.27/−2.17, Cohen’s *d* = 0.06, *p* < 0.001), as well as FAZ area increase (converter: EMD = 0.01, 95%CI = 0.03/0.18, Cohen’s *d* = 0.70, *p* = 0.005; non-converter: EMD = 0.12, 95%CI = 0.9/0.15, Cohen’s *d* = 1, *p* < 0.001). Only patients non-converting to dementia showed VD reduction in DCP (EMD = −4.20, 95%CI = −6.02/−2.37, Cohen’s *d* = 0.47, *p* < 0.001). VD of CC did not show significant differences in any of the groups (Table 3).

**Table 3:** Comparison in SD-OCT and OCTA parameters among non-converters vs. converters at baseline vs. follow-up.

	Baseline		Follow-up		Non-converters follow-up vs. baseline		Converters follow-up vs. baseline	
	Non-converters	Converters	Non-converters	Converters	EMD	*P*-value	EMD	*P*-value
SD-OCT (μM)								
GCC average	92.7 ± 11	91.5 ± 6.4	88.8 ± 11	86.7 ± 6.4	−3.8	0.04	−4.79	<0.001
RNFL average	95.6 ± 9.4	96.6 ± 7.1	92.3 ± 9.3	91.9 ± 7	−4.3	<0.001	−4.71	<0.001
OCTA (%)								
SCP whole	45.7 ± 4.9	44.6 ± 4.1	42.6 ± 4.9	41.7 ± 4.2	−3.19	0.001	−2.93	0.005
DCP whole	46.6 ± 5.3	43.6 ± 7.4	42.6 ± 4.4	41.4 ± 7.4	−4.2	<0.001	−2.22	0.26
CC whole	69.8 ± 3.9	70.6 ± 5.3	69.5 ± 4.5	70.4 ± 5.5	−0.3	0.6	−0.20	0.72
RPC whole	46.4 ± 4.8	46.7 ± 5.7	43.2 ± 5.1	42.3 ± 5.8	−3.22	<0.001	−4.40	<0.001
FAZ area	0.3 ± 0.1	0.3 ± 0.2	0.4 ± 0.1	0.4 ± 0.1	0.12	<0.001	0.01	0.005

SD-OCT, spectral domain optical coherence tomography; EMD, estimated mean difference; GCC, ganglion cell complex; RNFL, retinal nerve fiber layer; OCTA, OCT angiography; SCP, superficial capillary plexus; DCP, deep capillary plexus; CC, choriocapillary plexus; RPC, radial peripapillary capillary; FAZ, foveal avascular zone.

Linear mixed models, *P* value < 0.05.

Mean reduction in MMSE score in converting patients was 6.6, in non-converters 0.91 (*p* < 0.001).

In whole patient group, MMSE reduction was related to a more severe GCC reduction (coeff. β = 0.10; *p* = 0.04) and a more severe VD reduction in RPC plexus (coeff. β = 0.14; *p* = 0.02). When patients were divided in two groups according to the occurrence of conversion to dementia over the follow-up, only in patients converting to dementia, we found a higher delta MMSE associated with a more severe VD reduction in RPC plexus (coeff. β = 0.21; *p* < 0.001).

## Discussion

AD is usually diagnosed in the stage of dementia, when advanced neurodegeneration and vascular damage have already occurred. An overwhelming body of evidence indicates that discovery of disease-modifying treatments for AD should be aimed at the pre-dementia clinical stage of AD, i.e., MCI, to limit the damage and prevent further disease progression (Shi et al., 2020). Therefore, it is important to discover feasible biomarkers for predicting and monitoring the disease course.

Neurovascular unit integrity is necessary in maintaining normal central nervous system function. It is increasingly recognized that cerebral vascular abnormalities are early and pivotal factors in cognitive impairment in AD. Retinal vascular abnormalities such as changes in VD and fractal dimensions, blood flow, foveal avascular zone, curvature tortuosity and arteriole-to-venule ratio, oxygen saturation and arterial vessel diameter were reduced in early-stage AD and MCI (Frost et al., 2013; Cheung et al., 2014; Feke et al., 2015; Szegedi et al., 2020; Shi et al., 2021). Moreover, ischemia leads to disturbed nutrient supply, induces oxidative stress and inflammatory activities, impedes Aβ clearance and/or alters amyloid-processing enzymes (Marchesi, 2011), all of which can contribute to neurodegenerative processes and cognitive decline. Studies have also proposed that reduced cerebral blood flow associated with insufficient Aβ clearance may precede the onset of clinical dementia (Wolters et al., 2017; Govindpani et al., 2019).

The present study indicates a clear vascular impairment in aMCI patients in the inner retina and supports the hypothesis that vascular changes may contribute to the onset and progression of AD (Shi et al., 2021).

Previous OCTA investigations in MCI patient were conflicting, with significantly decreased VD only in the SCP (Zhang et al., 2019) or only in DCP (Wu et al., 2020) or in both (Yeh et al., 2022).

These conflicting data may find an explanation in the definition of MCI, which represents a continuum of cognitive decline between “normal aging” and dementia. Therefore, a wide and heterogeneous range of cognitive impairment is possible in MCI.

Despite many effort, further studies are need to identify a biomarker able to diagnose and monitor clinical progression of dementia, even if a recent study developed a multiregression framework to improve diagnostic ability of OCT to discriminate MCI and Alzheimer’s disease (Chua et al., 2022).

In our cohort, only patients non-converting to dementia showed a significantly VD reduction in DCP and a lower VD reduction in the same plexus was observed in converters vs. non-converters.

We hypothesize that DCP VD, which is composed of small and more vulnerable capillaries, starts reducing in the beginning of the neurodegenerative process in a more copious and evident way due to its initial increase. VD, indeed, is increased in preclinical AD patients probably for an early marked inflammatory response (Alber et al., 2020; van de Kreeke et al., 2020). With the progression of the disease and the consequent clinical evolution from preAD to MCI, and, at the end to overt dementia, the phenomenon becomes less evident due to the progressive depletion of VD (i.e., ceiling effect).

This hypothesis of a massive DCP VD depletion in the beginning of the disease, becoming less evident with its progression, is supported by meta-analysis data reporting that DCP VD of AD compared to MCI patients did not reach a statistical significant difference (Yeh et al., 2022).

The increase of the FAZ area in all patients, showing a large effect size (Cohen’s *d* = 0.99), together with the absence of association between FAZ area changes in converters and non-converters, support the hypothesis that the most sensitive and precocious vascular alteration in cognitive decline is the FAZ enlargement, but at the same time it is not specific enough to track the progression of the cognitive impairment.

Indeed, FAZ enlargement in literature has been already reported not only in MCI and AD but also in preclinical AD patients (Bulut et al., 2018; O’Bryhim et al., 2018; Yoon et al., 2019; Hui et al., 2021). On the other hand, also no significative differences in FAZ have been reported in AD, MCI and in cognitively healthy individuals with preclinical AD or with high genetic risk of AD (Zhang et al., 2019; van de Kreeke et al., 2020; López-Cuenca et al., 2021).

The discrepancy among all these studies may be due to many potential confounders including the different cohort of patients and different image processing.

Even if there is no conclusive evidence supporting the association between AD and impaired peripapillary vascularity. Lahme et al. (2018) reported a reduction of the peripapillary VD in patients with clinical AD. Montorio et al. (2022) reported a reduced VD in papillary region in preperimetric glaucoma and MCI, highlighting the common neurodegenerative process.

In our cohort a higher VD reduction in RPC plexus was found in converters vs. non-converters associated to higher GCC reduction. Both these parameters correlate with MMSE changes in the whole patient group, but only VD reduction in RPC plexus correlated to MMSE in converters. This data supports the role of RPC plexus as an index of neurodegeneration.

Our study has some limitations, in particular the low number of patients investigated, which might have limited the sensitivity towards smaller effect sizes. The main strength of the study is the longitudinal design, that might help shedding some light in the field of AD conversion prediction in MCI patients.

Further standardized studies with larger sample sizes and longer duration are warranted to adequately elucidate the role of retinal vascularization in cognitive aging and to determine if OCTA can be used as a biomarker for MCI and conversion to AD.

## Data availability statement

The raw data supporting the conclusions of this article will be made available by the authors, without undue reservation.

## Ethics statement

The studies involving human participants were reviewed and approved by Ethics Committee from Institutional Review Board of the University of Naples “Federico II” (protocol number: 142/19). The patients/participants provided their written informed consent to participate in this study. Informed consent has been obtained from all participants in the present study.

## Author contributions

CCr, GC, RL, VM, and CCo contributed to conception and design of the study. AC and ES organized the database. AC, DM, and MMo performed the statistical analysis. CCr wrote the first draft of the manuscript. CCr, DM, GC, and MMi wrote sections of the manuscript. DM, GC, and MMi collected data. All authors contributed to manuscript revision, read, and approved the submitted version.

## Conflict of interest

AC has received research grants from Almirall, research grants from ECTRIMS-MAGNIMS and honoraria from Almirall, Biogen, Roche Sanofi-Genzyme and Novartis. MMo has received research grants from ECTRIMS-MAGNIMS, UK MS Society, and Merck; and honoraria from Biogen, Merck, Roche, and Sanofi-Genzyme.

The remaining authors declare that the research was conducted in the absence of any commercial or financial relationships that could be construed as a potential conflict of interest.

## Publisher’s note

All claims expressed in this article are solely those of the authors and do not necessarily represent those of their affiliated organizations, or those of the publisher, the editors and the reviewers. Any product that may be evaluated in this article, or claim that may be made by its manufacturer, is not guaranteed or endorsed by the publisher.
