# Corrigendum: A two-year longitudinal study of retinal vascular impairment in patients with amnestic mild cognitive impairment

**DOI:** 10.3389/fnagi.2023.1197979

**Published:** 2023-04-20

**Authors:** Chiara Criscuolo, Gilda Cennamo, Daniela Montorio, Antonio Carotenuto, Miriana Migliaccio, Marcello Moccia, Elena Salvatore, Roberta Lanzillo, Ciro Costagliola, Vincenzo Brescia Morra

**Affiliations:** ^1^Reproductive and Odontostomatological Sciences, Department of Neurosciences, University of Naples “Federico II”, Naples, Italy; ^2^Public Health Department, Eye Clinic, University of Naples “Federico II”, Naples, Italy

**Keywords:** OCTA, aMCI, Alzheimer's disease, longitudinal study, mini mental state examination

In the published article, author names were incorrectly written as “Criscuolo Chiara^1*†^, Cennamo Gilda^2†^, Montorio Daniela^1^, Carotenuto Antonio^1^, Migliaccio Miriana^1^, Moccia Marcello^1^, Salvatore Elena^1^, Lanzillo Roberta^1*^, Costagliola Ciro^1†^ and Brescia Morra Vincenzo^1†^.”

The correct spelling is Chiara Criscuolo^1*†^, Gilda Cennamo^2†^, Daniela Montorio^1^, Antonio Carotenuto^1^, Miriana Migliaccio^1^, Marcello Moccia^1^, Elena Salvatore^1^, Roberta Lanzillo^1*^, Ciro Costagliola^1†^ and Vincenzo Brescia Morra^1†^.

In the published article, author names were incorrectly written in Author Contribution section as “CrC, CG, LR, BV, and CoC contributed to conception and design of the study. CA and SE organized the database. CA, MD, and MoM performed the statistical analysis. CrC wrote the first draft of the manuscript. CrC, MD, CG, and MiM wrote sections of the manuscript. MD, CG, and MiM collected data. All authors contributed to manuscript revision, read, and approved the submitted version”.

The correct spelling is CCr, GC, RL, VB, and CCo contributed to conception and design of the study. AC and ES organized the database. AC, DM, and MMo performed the statistical analysis. CCr wrote the first draft of the manuscript. CCr, DM, GC, and MMi wrote sections of the manuscript. DM, GC, and MMi collected data. All authors contributed to manuscript revision, read, and approved the submitted version.

In the published article, author names were incorrectly written in Conflict of interest section as “CA has received research grants from Almirall, research grants from ECTRIMS-MAGNIMS and honoraria from Almirall, Biogen, Roche Sanofi-Genzyme and Novartis. MoM has received research grants from ECTRIMS-MAGNIMS, UK MS Society, and Merck; and honoraria from Biogen, Merck, Roche, and Sanofi-Genzyme. The remaining authors declare that the research was conducted in the absence of any commercial or financial relationships that could be construed as a potential conflict of interest”.

The correct spelling is AC has received research grants from Almirall, research grants from ECTRIMS-MAGNIMS and honoraria from Almirall, Biogen, Roche Sanofi-Genzyme and Novartis. MMo has received research grants from ECTRIMS-MAGNIMS, UK MS Society, and Merck; and honoraria from Biogen, Merck, Roche, and Sanofi-Genzyme. The remaining authors declare that the research was conducted in the absence of any commercial or financial relationships that could be construed as a potential conflict of interest.

The authors apologize for this error and state that this does not change the scientific conclusions of the article in anyway. The original article has been updated.

